# Clinicopathologic analysis of progressive non-fluent aphasia and corticobasal degeneration: Case report and review

**DOI:** 10.1590/S1980-57642011DN05020013

**Published:** 2011

**Authors:** Paulo Roberto de Brito-Marques, Roberto José Vieira-Mello, Luciano Montenegro, Maria de Fátima Vasco Aragão

**Affiliations:** 1 Behavioral Neurology Unit, Department of Neurology, Faculty of Medical Sciences, University of Pernambuco, Recife PE, Brazil; 2 Department of Pathology, Health Sciences Center, Federal University of Pernambuco, Receife PE Brazil; 3 Centro de Diagnóstico Multimagem, Recife PE, Brazil

**Keywords:** progressive non-fluent aphasia, neuropsychology, neuroimaging, neuropathology, and corticobasal degeneration

## Abstract

**Objective:**

To investigate progressive non-fluent aphasia and histopathologically-proven corticobasal degeneration.

**Methods:**

We evaluated symptoms, signs, neuropsychological deficits, and radiology data longitudinally, in a patient with autopsy-proven corticobasal degeneration and correlated these observations directly to the neuroanatomic distribution of the disease.

**Results:**

At presentation, a specific pattern of cognitive impairment was evident with an extreme extrapyramidal motor abnormality. Follow-up examination revealed persistent impairment of praxis and executive functioning, progressive worsening of language performance, and moderately preserved memory. The motor disorder manifested and worsened as the condition progressed. Many of the residual nerve cells were ballooned and achromatic with eccentric nuclei. Tau-immunoreactive pathology was significantly more prominent in neurons in the frontal and parietal cortices and dentate nuclei than in temporal neocortex, hippocampi and brainstem.

**Conclusion:**

The clinical diagnosis of progressive non-fluent aphasia secondary to corticobasal degeneration hinged on a specific pattern of impaired cognition as well as an extrapyramidal motor disorder, reflecting the neuroanatomic distribution of the disease in frontal and anterior temporal cortices and the dentate nuclei.

## Abstract (pt)

**Objetivo:**

Investigar o quadro clínico de afasia progressiva não-fluente e confirmação histopatológica de degeneração córtico-basal.

**Métodos:**

Foram avaliadas as alterações clínicas, neuropsicológicas e de neuroimagem, durante todo o curso clínico da doença. O diagnóstico de degeneração córtico-basal foi confirmado por estudo histopatológico. Essas observações foram diretamente relacionadas com a distribuição anatômica da doença.

**Resultados:**

Foi observada uma forma específica de prejuízo cognitivo associada com importante alteração extrapiramidal. Durante o curso clínico, surgiram apraxia e disfunção executiva, piora progressiva da linguagem e memória moderadamente preservada. As alterações extrapiramidais pioraram progressivamente à rigidez universal e postura distônica. As reações de imunohistoquímica para a proteína *tau* foram significativamente mais proeminentes nos neurônios residuais de aspecto baloniformes e acromáticos do córtex frontal e parietal e núcleo denteado do cerebelo do que nos do neocórtex temporal, hipocampo e tronco cerebral.

**Conclusão:**

O diagnóstico clínico de afasia progressiva não-fluente quando evolui para degeneração córtico-basal deve apresentar uma forma específica de prejuízo cognitivo, incluindo as alterações motoras, refletindo uma distribuição neuroanatômica da doença no córtex frontal e temporal anterior e no núcleo denteado do cerebelo.

## Introduction

The Consensus Criteria for FTLD distinguishes three variants of FTLD which reflect the predominant locus of the pathology: frontal variant frontotemporal dementia (FTD), semantic dementia (SD) and progressive non-fluent aphasia (PNFA).^1^ FTD is characterized by alterations in behavior, personality and executive functions. The core diagnostic features include insidious onset and gradual progression of insight loss, early decline in social interpersonal conduct, and regulation of personal conduct as well as early emotional blunting. Two other clinical subtypes of FTLD have been characterized for the most prominent symptoms of the language dysfunction. PNFA is a disorder of expressive language, including non-fluent spontaneous speech with agrammatism or phonemic paraphasias or anomia. The core diagnostic features of the SD include progressive, fluent, empty spontaneous speech, loss of word meaning manifested by impaired naming and comprehension, semantic paraphasias and/or perceptual disorders along with agnosia for faces and objects. Behavioral changes in SD are more common than in PNFA.^2^ FTDL, corticobasal degeneration (CBD), and progressive supranuclear palsy (PSP) however, have overlapping clinical features, and most cases are classified as progressive non-fluent aphasia. The predictability of tau-positive pathology from a CBD or PSP-like presentation is good.^3^

Corticodentatonigral degeneration with neuronal achromasia was first described in 1968 by Rebeiz and et al., and is a neurodegenerative disease with both motor and cognitive dysfunctions. The diagnosis is probably underestimated because of the heterogeneity of clinical features and overlapping of symptoms and pathological findings with other neurodegenerative diseases. The most characteristic initial motor symptoms are akinesia, rigidity, and apraxia. Dystonia and alien limb phenomena are frequently observed. There is often a parkinsonian picture with failure or lack of efficacy of dopaminergic medical therapy. Cognitive decline, prompting the diagnosis of dementia, may be the most common misdiagnosed presentation of CBD. Neuroimaging and electrophysiological studies may help in reaching the diagnosis but are not specific. Pathology is characterized by asymmetric frontoparietal neuronal loss and gliosis with ballooned, achromatic cortical neurons, nigral degeneration, and variable subcortical involvement.^4,5^

In this clinicopathologic report, we examined an unusual case of autopsy-proven CBD, showing progressive aphasia and falls as the first symptoms. The CBD diagnosis was confirmed by histopathologic abnormalities.

## Methods

The study was approved by the local Research Ethics Committee of Oswaldo Cruz Hospital of University of Pernambuco - Brazil. A 67-year-old female patient was diagnosed with corticobasal degeneration according to immunohistochemical reaction with antibodies against tau and ubiquitin proteins. Neuropsychological assessment was based on the Mini-Mental State Examination (MMSE),^6^ ANAD,^7^ Token-Test,^8^ and the Trail Making Test.^9^ The patient was also submitted to neuroimaging studies including computed tomography (CT) and magnetic resonance imaging (MRI), and to brain single photon emission computed tomography (SPECT) analysis. The neuropathological examination was performed according to the European Concerted Action on Pick’s Disease.^10^ The brain was fixed in 10% formalin for 4 weeks and cut into coronal sections. Fragments of the middle frontal, upper temporal, middle temporal, inferior temporal, inferior parietal gyri, hippocampus, brainstem and cerebellum, were routinely processed and stained with hematoxylin-eosin, Paz’s silver, Masson, Wölcke’s myelin and Bielschowsky methods. Immunohistochemical reaction with antibodies against tau and ubiquitin proteins was carried out.

## Case report

When the female patient first presented at our service in May 1996, she was 67 years old with a brief history of hyperthyroidism, maintained on methimazole, and complained of a two-year history of progressive difficulties speaking, and episodes of unexplained falls. She had nine years of schooling, and no family history of dementia, parkinsonianism or other neurological illness suggestive of motor neuron disease was reported.

On initial examination, the patient was aware, alert, and cooperative. She was right-handed and well-oriented in terms of place, but less concerning time. She scored 24/30 on the MMSE, showed disturbances of attention on the Bells test (24/35) and on the Trail-making test (especially on B type). The Token test was normal. Category fluency was assessed for both animal names and letters, although letter fluency was assessed using a number of “P” words. The average number of novel target words generated in 90 seconds was scored. The patient had major difficulties for both verbal and semantic fluencies, 1\18 and 8\18, respectively. There was a motor disorder typically characterized by initially impaired speech output, slow speech rate, and prolonged intervals between syllables and words. She had difficulty understanding complex subjects, retelling stories in a logical sequence, and memorizing both names and images. Her logical memory was consistent with her age. In a test of visual discrimination, she committed errors of negligence, mirroring, and denial of stimuli. She showed agnosia in identifying familiar objects, and constructive apraxia in copying Rey’s figure.

Examination of the oculomotor nerves showed downward vertical gaze paresis. She exhibited Myerson’s sign and increased reflexes of the *orbicularis oris*. She did not develop parkinsonian symptoms, such as tremor and cog-wheel rigidity. Deep tendon reflexes were 4MM 4+, with a brisk patellar reflex, but presented no clonus. The patient exhibited a flexor plantar reflex and her gait was unstable with a tendency to fall backwards.

On admission, the patient’s complete blood count, basic metabolic panel, folic acid, vitamin B12, thyroid function tests, calcium, phosphorus, magnesium, and cortisol levels were normal. Ultrasound of the carotid arteries was also normal. The CT of the brain showed diffuse brain atrophy without white matter disease, compatible with normal aging. The MRI of the brain showed an interuncal distance of 3 cm and loss of volume in the parahippocampal gyri. The SPECT of the brain showed hypoperfusion of the frontal lobes bilaterally and the left anterior temporal lobe.

Two years after diagnosis, the patient was apathetic, but noticed movement of people in the house, from the television, etc. She was still responsive to some verbal commands, and was alert and well-oriented, but to a lesser degree concerning place and time. She scored 20/30 on the MMSE and exhibited marked dysarthria, and plastic hypertonia with flexion of the neck, trunk, and limbs. She had progressive generalized parkinsonian cogwheel rigidity on passive movement. Her gait was wide-based, with her body tilted forward, mild festination, and a tendency to fall back, evolving into abasia and major difficulty walking. The SPECT brain scan revealed a bilateral moderate to severe frontal supplementary area and a mild to moderate prefrontal and anterior parietal lobe hypoperfusion, particularly on the left side.

**Figure 1:**
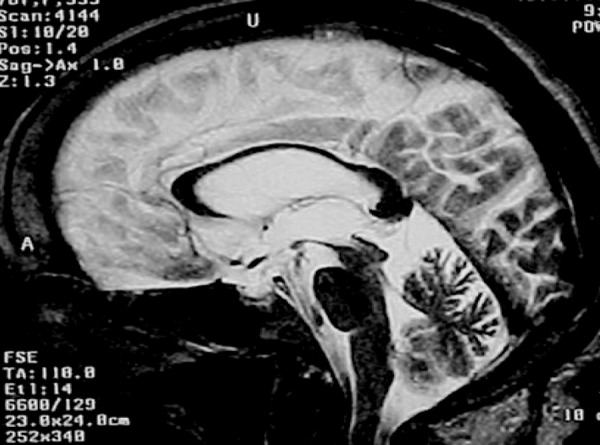
Sagittal T2-weighted images shows marked atrophy of the superior colliculus (midbrain) and marked atrophy of the body of the corpus callosum.

Three years after diagnosis, MRI showed reduction of the substantia nigra *pars compacta*. The superior colliculi were smaller than the inferior colliculi, and the midbrain diameter measured 11 mm, and signs of cerebellar atrophy were evident (Figure 1).

After six years, the patient remained constantly in the supine position, with eyes open and no response to verbal commands. She was anarthric. Both upper limbs were flexed and adducted with intense rigidity. The right hand was closed, while the left was extended. She had significant flexion of the lower extremities, adduction of the thighs, and internal rotation of the left foot. Deep tendon reflexes were increased. Plantar reflex showed extension in the left foot and extension with fanning of the toes on the right foot. She had urinary and fecal incontinence. She died at the age of 75, after a total clinical course of 8 years from diagnosis. Her death was attributed to an infection.

**Figure 2:**
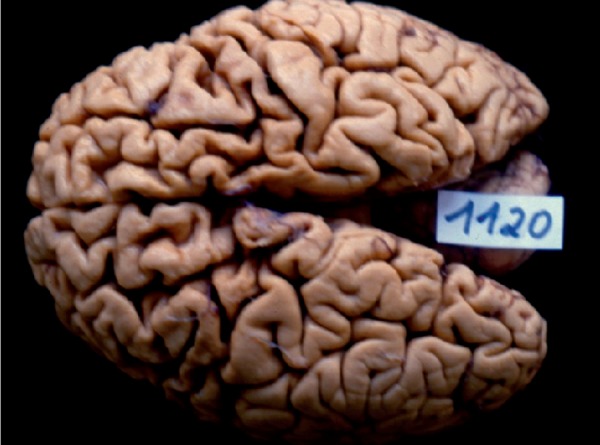
Gross analysis shows significant moderate to severe bilateral frontotemporal atrophy.

**Figure 3:**
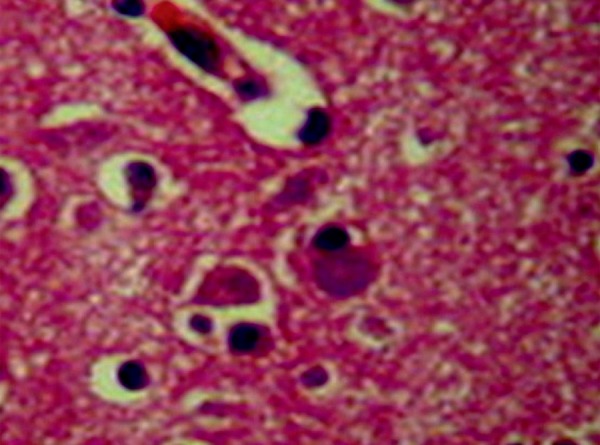
Histopathology with Hematoxylin and Eosin stain in frontal lobe reveals ballooning neurons.

On macroscopic pathology (Figure 2), the brain weighed 750 grams before fixation. The brain had significant moderate to severe bilateral frontotemporal atrophy, and intense bilateral atrophy of the dentate and ambiguous nuclei on the cerebellum. On microscopy (Figure 3), histopathologic abnormalities were most prominent in frontal and anterior temporal regions, and dentate nuclei, although variable histopathology was also present in other brain regions. There was neuronal depopulation (ND), tau protein in arc (TP) and ubiquitin in rings (U) within ballooning neurons (BN). Senile plaques (SP) were found in the frontal gyri (ND, BN, SP, TP, U), motor cortex (ND, BN), sensory cortex (BN), temporal gyri (BN, SP, TP), hippocampus (BN, U, SP), nuclei ambiguous (BN), dentate nuclei of the cerebellum (BN, gliosis), mesencephalon and pons (neuronal depigmentation, TP, U). However, no Lewy bodies were found.

## Discussion

Our findings support growing evidence that CBD presents with cognitive difficulties as prominent as its motor deficits. The cognitive picture emerging from our study highlights a characteristic impairment of executive, social, language, and visuospatial functioning, but spared episodic memory, although mild to moderate disease. This pattern of selective cognitive impairment appeared to predict the anatomic distribution of the pathology, with relatively prominent frontal and anterior temporal disease in addition to ambiguous and dentate nuclei pathology, yet modest involvement of hippocampus and brainstem. We concluded that the characteristic clinical features of CBD reflected cognitive impairment according to progressive non-fluent aphasia and subsequent corticobasal syndrome, as well as the motor disorder itself, all of which corresponded with histopathologic diagnosis. We will now discuss the clinical features consistent with a pathologic diagnosis of CBD.

Our patient exhibited virtually all extrapyramidal motor features prior to death, where the same spectrum of motor difficulty described in the literature for CBD was observed.^3,5,11-14^ The patient had no asymmetric limb rigidity, alien hand phenomena, or myoclonus. According to the neuroanatomic basis for these motor difficulties, the typical motor and atypical cognitive pattern of impairment underlines that this study represents an unusual sample of a case with CBD that included mild pyramidal syndrome during the year before death.

Patients with CBD are often said to have few cognitive deficits.^3,5^ Cognitive symptoms are featured less prominently than are motor deficits at presentation, according to this view.^5^ Apraxia is believed to be a hallmark of CBD^15^ which is observed in frontal and parietal brain regions.^3,16,17^ In the present case, the reverse order of occurrence was found whereby cognitive deficits preceded motor difficulties as the disease evolved clinically. The profile of cognitive difficulty appeared to correspond to the distribution of the disease with language disorder as a first symptom. The patient complained about new things, and dressing and writing difficulties were common. Neurologic examination revealed ideomotor, dressing and important constructive apraxia. In this case, these neurologic findings were consistent with an imaging study associating with greater SPECT defects in the bilateral supplementary motor area than in prefrontal and anterior parietal lobes. On the other hand, MRI showed moderate atrophy of the middle portion of the corpus callosum, besides marked atrophy of the superior colliculus. From an imaging perspective, atrophy of the corpus callosum with middle predominance was present in CBD, and this atrophy is associated with cognitive impairment and cerebral cortical hypometabolism with hemispheric asymmetry. Atrophy of the corpus callosum may reflect the severity of the disconnection between cortical regions, and this could be an important factor in the development of cerebral cortical dysfunction in CBD.^8,19,20^

Significant executive difficulty in the case reported was related to prominent frontal pathology in CBD. There are few reports of executive dysfunction in autopsy-confirmed cases of CBD, but executive difficulties such as impaired planning, poor inhibitory control, and limited mental search have been described in patients with clinically-diagnosed CBS.^21,22^ In the current case, the patient and their caregivers rarely complained about executive difficulties. However, deficits in mental search were present in this patient at initial examination. This was confirmed by neuropsychological assessments showing significant deficits on measures of category naming fluency. These findings should be interpreted cautiously because deficits on verbally mediated measures might be due in part to language difficulty. However, in the test of visual discrimination, the patient had significant difficulty constructing a perceptual representation from vision (apperception) and mapping of perceptual representation onto stored perception of the object’s functions and associations. These findings were interpreted as cortical dysfunction.

A disorder of social behavior and personality has been described in patients with pathologically-confirmed CBD, including bizarre behavior, personality change, and apathy.^16^ The patient in this case had deficits in mental search along with characteristic traits such as industriousness, seriousness and inflexibility, and apathy throughout the disease course, but no depression.

According to the literature,^16,21,23-30^ frontal and parietal involvement may also play a role in the pattern of language and apraxia impairment seen in CBD. In the present study, the patient frequently complained of speech and handwriting difficulties. Neurologic examination confirmed the common occurrence of a motor disorder typically characterized with initially impaired speech output, slow speech rate, prolonged intervals between syllables and words, as apraxia of speech, but language impairment increased markedly evolving to anarthria as the disease progressed. Neuropsychological evaluation showed a mild deficit in naming and single-word comprehension at presentation. The grammatical comprehension deficit observed was due in part to a limitation in executive resources needed to understand a complex sentence. Clinical features and progressive course of this language disorder were suggestive of PNFA.

Visuospatial difficulties also are reported in patients with pathologically confirmed CBD.^27^ Visuospatial complaints and signs were common in the present case which exhibited visuospatial disorder in addition to visual agnosia. Neuropsychological measures of visual construction showed a significant impairment. Although these tasks had a motor component, spatial errors seen were suggestive of parietal impairment. In this state, the simplest of geometric forms and patterns were not copied. Her second copy of a Rey’s figure was substantially altered. The present study also emphasized the selective nature of cognitive impairment. On clinical memory examination and neuropsychological testing, she demonstrated significant deficit in incidental memory and moderate deficit in logical memory during the course of CBD.

In more than half CBD cases, the affected neurons and adjacent glial have been shown to be filled with a particular configuration of *tau* protein.^27^ The remaining cases have shown *tau* deposition more closely resembling that of PSP, Alzheimer pathology, or nonspecific cell loss with replacement gliosis.^27^ There are no ballooned neurons on the PSP.^31^ According to previous studies,^17,32,33^ the pathology is present throughout frontal, parietal, and temporal cortical regions as well as the hippocampus, basal ganglia, and substantia nigra. On macroscopic pathology study, this case showed atrophic lesions to frontotemporal lobes, but histopathologic microscopy of these lobes revealed *tau*-positive inclusions in residual cell bodies of NB with eccentric nuclei, some astrocytic plaques, ND and atrophy. Ultrastructural study was not performed. The most severe atrophy and pathology were observed in the frontal and anterior temporal cortex presenting ND, BN, SP, TP immunoreactivity and intense bilateral atrophy in the dentate and ambiguous nuclei showing very few BN and gliosis, whereas significantly less atrophy and tau immunoreactivity was seen in hippocampal and superior parietal regions.

However, the clinical diagnosis of CBD is very challenging. According to Ling et al., of 19 pathologically-confirmed corticobasal degeneration cases, only five had been diagnosed correctly in life (sensitivity=26.3%). On the other hand, from among 21 cases with a clinical diagnosis of CBD, only five had corticobasal degeneration pathology, giving a positive predictive value of 23.8%.^34^

In conclusion, this study demonstrated that the FTLD, PSP, and CBD had overlapping clinical features. However, some syndromes were better correlated with specific pathologies than others, as PNFA and subsequent corticobasal syndrome. MRI study of the brain showed an interuncal distance of 3 cm and loss of volume in the parahippocampal gyri, reduction of the substantia nigra pars compacta. The superior colliculi were smaller than the inferior colliculi and there were signs of cerebellar atrophy. SPECT of the brain showed hypoperfusion of the frontal lobes bilaterally, and of the left anterior temporal lobe. Gross pathology revealed moderate to severe bilateral frontal and anterior temporal lobe degeneration. The diagnosis of CBD was reached based on histopathologic study only, showing residual nerve cells with ballooning aspect, eccentric nuclei and achromasia, cytoplasmic vacuolation in the frontal and temporal cortex, and dentate, as well as ambiguous nuclei. In order to enable future treatment of many of these neurodegenerative diseases, further studies, both clinicopathological as well as those utilizing biomarker techniques, are needed to improve diagnostic sensitivity.

## Figures and Tables

**Figure 4:**
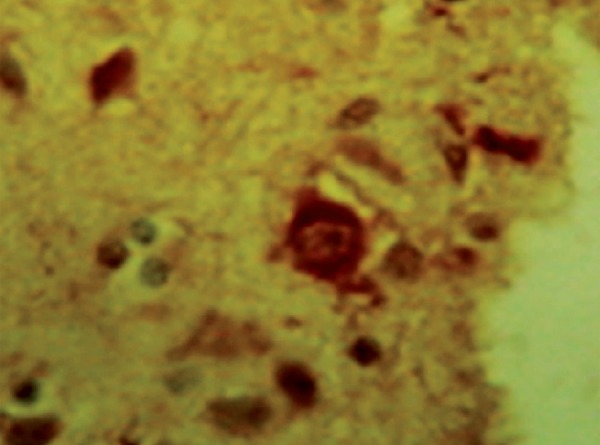
Immunohistochemistry reaction shows antibodies against ubiquitin proteins in rings in frontal lobe and also neuronal loss.

**Figure 5:**
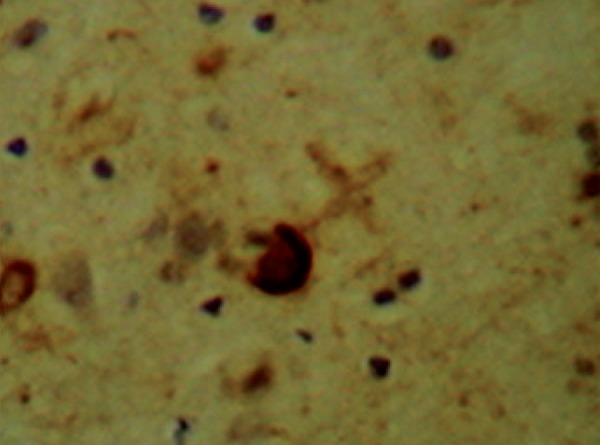
Immunohistochemistry reaction shows antibodies against tau proteins in arc in frontal lobe and also neuronal loss.

## References

[1] Neary D, Snowden JS, Gustafson L (1998). Frontotemporal lobar degeneration. A consensus on clinical
diagnostic criteria. Neurology.

[2] Gorno-Tempini ML, Dronkers NF, Rankin KP (2004). Cognition and anatomy in three variants of primary progressive
aphasia. Ann Neurol.

[3] Josephs KA, Petersen RC, Knopman DS (2006). Clinicopathologic analysis of frontotemporal, corticobasal
degenerations and PSP. Neurology.

[4] Stover NP, Watts RL (2001). Corticobasal degeneration. Sem Neurol.

[5] Rebeiz JJ, Kolodny EH, Richardson EP Jr (1968). Corticodentatonigral degeneration with neuronal achromasia: a
progressive disorder of late adult life. Trans Am Neurol Assoc.

[6] Folstein MF, Folstein SE, McHugh PR (1975). Mini-mental state: a practical method for grading the cognitive
state of patients for the clinician. J Psychiat Res.

[7] Joanette Y, Arlette P, Bernadette S, Brito-Marques PR (1995). Avaliação neuro-psicológica adequada
às demências. Arq Neuropsiquiatr.

[8] DeRenzi E, Faglioni P (1978). Normative data and screening power of a shortened version of the
Token Test. Cortex.

[9] Reitain R (1958). Validity of the trail making test as an indicator of organic
brain damage. Percept Mot Skills.

[10] European Concerted Action on Pick.s Disease (ECAPD) (1998). Consortium: provisional clinical and neuropathological criteria
for the diagnosis of Pick's disease. Eur Neurol.

[11] Rinne JO, Lee MS, Thompson PD, Marsden CD (1994). Corticobasal degeneration: a clinical study of 36
cases. Brain.

[12] Wenning GK, Litvan I, Jankovic J (1998). Natural history and survival of 14 patients with corticobasal
degeneration confirmed at postmortem examination. J Neurol Neurosurg Psychiatry.

[13] Gibb WRG, Luthert PJ, Marsden CD (1989). Corticobasal degeneration. Brain.

[14] Boeve BF, Maraganore DM, Parisi JE (1999). Pathologic heterogeneity in clinically diagnosed corticobasal
degeneration. Neurology.

[15] Litvan I, Bhatia KP, Burn DJ (2003). SIC task force appraisal of clinical diagnostic criteria for
parkinsonian disorders. Mov Disord.

[16] Grimes DA, Lang AE, Bergeron CB (1999). Dementia as the most common presentation of corticobasal
ganglionic degeneration. Neurology.

[17] Murray R, Neumann M, Forman MS (2007). Cognitive and motor assessment in autopsy-proven corticobasal
degeneration. Neurology.

[18] Yamauch H, Fukuyama H, Nagahama Y (1998). Atrophy of the corpus callosum, cortical hypometabolism, and
cognitive impairment in corticobasal degeneration. Arch Neurol.

[19] Brito-Marques PR, Vieira de Melo R, Montenegro L (2000). Classic Pick's disease type with ubiquitina-positive and
tau-negative inclusions: report of a case. Arq Neuropsiquiatr.

[20] Brito-Marques PR, Vieira de Mello R, Montenegro L (2002). Frontoparietal cortical atrophy with gliosis in the gray matter
of cerebral cortex. Arq Neuropsiquiatr.

[21] Massman PJ, Kreiter KT, Jankovic J, Doody RS (1996). Neuropsychological functioning in cortical-basal ganglionic
degeneration: differentiation from Alzheimer's disease. Neurology.

[22] Pillon B, Blin J, Vidailhet M (1995). The neuropsychological pattern of corticobasal degeneration:
comparison with progressive supranuclear palsy and Alzheimer's
disease. Neurology.

[23] Graham NL, Bak T, Hodges JR (2003). Corticobasal degeneration as a cognitive disorder. Mov Disord.

[24] Kertesz A, Hudson L, Mackenzie IR, Munoz DG (1994). The pathology and nosology of primary progressive
aphasia. Neurology.

[25] Kertesz A, Martinez-Lage P, Davidson W, Munoz DG (2000). The corticobasal degeneration syndrome overlaps progressive
aphasia and frontotemporal dementia. Neurology.

[26] Mimura M, White RF, Albert ML (1997). Corticobasal degeneration: neuropsychological and clinical
correlates. J Neuropsychiatry Clin Neurosci.

[27] Riley DE, Lang AE, Lewis A (1990). Cortico-basal ganglionic degeneration. Neurology.

[28] Tang-Wai DF, Josephs KA, Boeve BF, Dickson DW, Parisi JE, Petersen RC (2003). Pathologically confirmed corticobasal degeneration presenting
with visuospatial dysfunction. Neurology.

[29] Josephs KA, Duffy JR (2008). Apraxia of speech and nonfluent aphasia: a new clinical marker
for corticobasal degeneration and progressive supranuclear
palsy. Curr Opin Neurol.

[30] Josephs KA, Duffy JR, Strand EA (2006). Clinicopathological and imaging correlates of progressive aphasia
and apraxia of speech. Brain.

[31] Freeman RQ, Giovannetti T, Lamar M (2000). Visuoconstructional problems in dementia: contribution of
executive systems functions. Neuropsychology.

[32] Mochizukia A, Ueda Y, Komatsuzaki Y, Tsuchiya K, Arai T, Shoji S (2003). Progressive supranuclear palsy presenting with primary
progressive aphasia: clinicopathological report of an autopsy
case. Acta Neuropathol.

[33] Forman MS, Zhukareva V, Bergeron CB (2002). Signature tau neuropathology in gray and white matter of
corticobasal degeneration. Am J Pathol.

[34] Ling H, O'Sullivan SS, Holton JL (2010). Does corticobasal degeneration exist? A clinicopathological
re-evaluation. Brain.

